# Lanthanide doped lead-free double perovskites as the promising next generation ultra-broadband light sources

**DOI:** 10.1038/s41377-022-00782-z

**Published:** 2022-04-19

**Authors:** Li Zhang, Mingjian Yuan

**Affiliations:** 1 Key Laboratory of Advanced Energy Materials Chemistry (Ministry of Education), Renewable Energy Conversion and Storage Center (RECAST), College of Chemistry, Nankai University, Tianjin, China; 2 Haihe Laboratory of Sustainable Chemical Transformations, Tianjin, China

**Keywords:** Inorganic LEDs, Organic LEDs

## Abstract

Efficient ultra-broadband emitter is realized by using lanthanide ion doping coupled with “DPs-in-glass composite” (DiG) structure. The synergy of self-trapped exciton together with the energy transition induce this ultra-broadband emission emerge.

## Abstract (web summary)

Efficient ultra-broadband emitter is realized by using multi-Ln-doped Cs_2_Ag(In/Bi)Cl_6_ lead-free double perovskite (DP) coupled with “DPs-in-glass composite” (DiG) structure. The synergy of self-trapped exciton together with the energy transition induces this ultra-broadband emission emerge.

## Graphical abstract

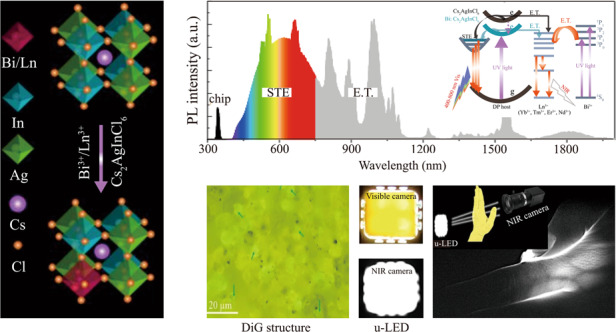

Ultra-broadband emitter is critical to advancing the applications of light sensing, spectrum analysis, and life sciences imaging, et al. With the development of high-capacity optical data communications and ultra-precision metrology^1,2^, efficient ultra-bandgap emission becomes particularly important. Traditional ultra-broadband light sources generally include halogen tungsten lamps (HTLs)^3^, super-luminescent diodes (SLDs)^4^, ultra-broadband semiconductor lasers (UBSLs)^5^, laser-driven light sources (LDLSs)^6^, super-continuum light sources (SCLSs)^7^, etc. However, many shortcomings still exist, such as spectral instability, high electrical consumption, short lifetime, substantial heat generation, and noncompactness. Hence, alternative ultra-broadband light sources with outstanding optical and structural properties are highly demanded.

Metal halide perovskites have attracted widespread attention due to their outstanding optoelectronic properties^8–10^, making them as the promising monochromatic bright emitters. However, toxicity and poor material stabilities of traditional lead perovskites impede their further commercialization^11^. Accordingly, lead-free halide double perovskites (DPs) have drawn increasing attention recently owing to their fascinating optical properties and excellent stabilities. In particular, lanthanide (Ln^3+^) ion doping to tailor the optical or electrical properties of DPs has been well documented, aiming for their applications in white LED, NIR-LED, scintillator, anti-counterfeiting, and X-ray detecting^12^. The progresses leverage the opportunity to realize ultra-broadband emission using Ln^3+^-doped DPs, which has never been explored.

Chen’s group here reported the pioneer work to realize ultra-broadband continuous emission from visible to near-infrared spectral region (400–2000 nm) in Cs_2_AgInCl_6_ DPs, by combining the self-trapped exciton (STE) and extra luminescence channel induced by Ln^3+^ doping^13^ (Fig. 1a). In particular, the Bi/Ln co-doped Cs_2_AgInCl_6_ (Bi/Ln (Ln = Nd, Yb, Er, Tm): Cs_2_AgInCl_6_) exhibit both visible STE and multiple NIR Ln^3+^ 4f-4f emissions under excitation^14^, which enables ultra-broadband emission (Fig. 1b). Energy transfer mechanism was proposed to explain the origin of the Ln^3+^ emission in Bi/Ln: Cs_2_AgInCl_6_ DPs. Notably, Bi^3+^ doping is critical to enabling Ln^3+^ emission, since Bi^3+^ doping can modulate the density of states at the band edge, break parity forbidden transition of STE states and promote exciton localization, giving rise to new optical channels at a lower energy level and promoting efficiency of STE emission^15^. Moreover, two intense absorptions transitions of Bi^3+^ were observed, which were ascribed to the ^1^S_0_ → ^1^P_1_ and ^1^S_0_ → ^3^P_1_ transition. The process effectively transfers energy to Ln^3+^ dopants, to enable multiple emission of 4f–4f transitions that resulted in NIR emissions^16^.

**Fig. 1:**
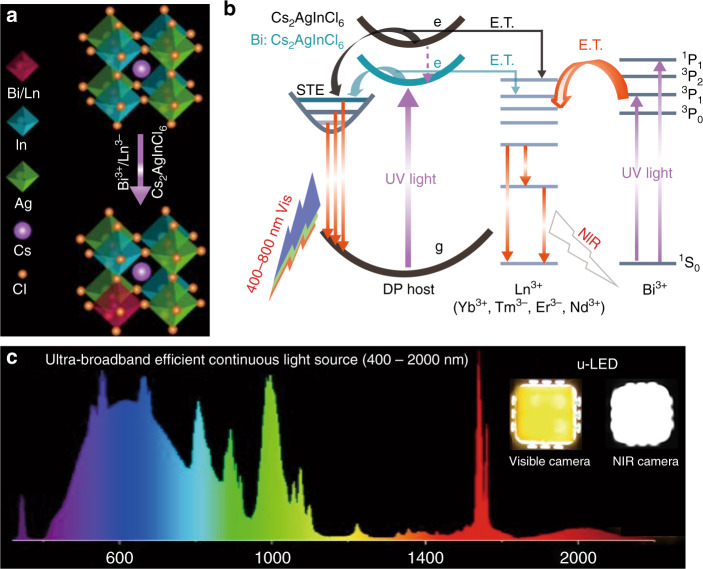
*u*-LED relying on the synergy of (STE) recombination and Ln^3+^ dopants’ 4f-4f transitions of the multi-Ln^3+^-DiG. **a** Structure diagram of Bi/Ln:Cs_2_AgInCl_6_ DPs. **b** The ultra-broadband emission mechanisms in Bi/Ln:Cs_2_AgInCl_6_ DPs. **c** PL spectrum of the u-LED device. The inset is the photographs of the multi-Ln^3+^-DiG u-LED by visible camera (yellow) and NIR camera (white)

The synergy of STE broadband emission (400–800 nm) and narrowband NIR emissions from Ln^3+^ (Yb^3+^, Tm^3+^, Er^3+^, and Nd^3+^) thus induce ultra-broadband continuous luminescence. As shown in Fig. 1, multiple Ln^3+^ activators need to be doped into DPs host, but the energy transfer and cross-relaxation processes among them typically led to the energy loss via non-radiative relaxation, resulting in quenched Ln^3+^ emissions in the multi-doped DPs^17^. To solve the problem, they constructed a unique DPs-in-glass (DiG) monolithic composite to confine different Ln^3+^ dopants and avoid their interaction. Specifically, Nd:Cs_2_AgInCl_6_, Yb/Er: Cs_2_AgInCl_6_ and Yb/Tm:Cs_2_AgInCl_6_ DPs were dispersed into an inorganic glass matrix by low temperature co-sintering. The above bottom-up strategy endows the prepared Ln^3+^-doped DiG with an improved PLQY of 40% and superior long-term stability.

The DiG was then coupled with commercial 350 nm UV chip to fabricate lighting devices, representing the record ultra-broadband light source covering spectral region from 400 to 2000 nm with full width at half maxima (FWHM) of ~365 nm (Fig. 1c). Furthermore, Chen et al. showcase the compact ultra-broadband LED’s (u-LED’s) applications in nondestructive spectroscopic analysis and multifunctional lighting^13^.

The brand-new strategy conceived by Chen et al. thus provides a powerful toolbox to tailoring multi-Ln^3+^-doped DPs to realize efficient ultra-broadband emitters. The strategy certainly will attract widespread attention from the whole community, and facilitate their application in various fields such as multi-functional lighting, optical communication, and nondestructive spectral analysis. The lanthanide-doped lead-free DPs thus represent a promising candidate for next generation ultra-broadband light sources.
